# Orofacial migraine and neurovascular orofacial pain—new insights into characteristics and classification

**DOI:** 10.22514/jofph.2024.033

**Published:** 2024-12-12

**Authors:** Shimrit Heiliczer, Yair Sharav, Rafael Benoliel, Yaron Haviv

**Affiliations:** ^1^Department of Oral Medicine, Sedation and Imaging, Hadassah Medical Center, Faculty of Dental Medicine, Hebrew University of Jerusalem, 91120 Jerusalem, Israel; ^2^Oral Medicine Unit, Oral and Maxillofacial Surgery Department, Tel Aviv Sourasky Medical Center, 64239 Tel Aviv, Israel; ^3^Rutgers School of Dental Medicine, Newark, NJ 07103, USA

**Keywords:** Orofacial migraine, Neurovascular orofacial pain, International classification of orofacial pain

## Abstract

Orofacial migraine (OFM) and neurovascular orofacial pain (NVOP) are both recognized as migraine-related entities affecting the facial and orofacial regions, according to the International Classification of Orofacial Pain (ICOP). However, the distinction between these two conditions and the question of whether NVOP should be considered a separate entity remain subjects of ongoing debate. The aim of this study is to compare the diagnostic characteristics of OFM and NVOP to reassess whether they should continue to be classified as two distinct diagnoses. The cohort comprised 75 patients, 12 males and 63 females, 40 were diagnosed as NVOP and 35 as OFM according to ICOP criteria. Patients were recruited from the tertiary orofacial pain clinic in Hadassah Medical Center between the years 2016 to 2023. NVOP and OFM patients did not differ in age, sex, pain intensity and other pain characteristics. However, OFM patients have significantly more cranial autonomic signs (36.4%) than NVOP patients (10.3%), and also more migraine symptoms such as nausea and photophobia. (68.6% *vs.* 41%) OFM patients reported significantly more awakening from sleep (52.9%) than NVOP patients (26.3%). Also, OFM pain was concomitant with headache in about two third of cases (66.7%), compared to only a third (30.8%) of NVOP cases. Most NVOP patients have pain that mimics toothache (85%), rarely detected in OFM (11.4%). The diagnostic features of OFM and NVOP indicate that there are many similarities between the two. But also, unique features that allows for separating OFM and NVOP into two distinct diagnostic entities, in accordance with the ICOP classification. Inclusion of patients with associated headaches enhanced this separation, and suggests expanding the definition of ICOP and include it under OFM and NVOP. At present there is justification to maintain the separate ICOP classifications of NVOP and OFM, particularly for research purposes.

## 1. Introduction

Presentations of primary headaches in the lower face have been recognized [1] and recently, were classified in the International Classification of Orofacial Pain, 1st edition (ICOP) [2]. The publication of ICOP endorse the interest in orofacial pain (OFP), resulting in a host of publication on this topic. The nature of pain, constant or attack-like was addressed [3] but the limitation of defining OFP as chronic due to its periodic short-lasting nature was emphasized [4, 5]. A series of articles conducted research in order to characterize patients with OFP according to ICOP [5, 6]. And orofacial migraine and other idiopathic non-dental facial pain syndromes were surveyed [7]. The ICOP classification was further explored, pointing to its limitations in fully capturing chronic OFP multifaceted nature and the complex interplay of biological, psychological and social factors [8]. Also, the association between OFP and depression or anxiety were extensively reviewed [9, 10].

Section 5 in ICOP defines “orofacial pains resembling presentations of primary headaches”. This section covers 4 main sub-sections; orofacial migraine, tension-type orofacial pain, trigeminal autonomic orofacial pain and neurovascular orofacial pain. Of interest to this article are the criteria for orofacial migraine (OFM) and neurovascular orofacial pain (NVOP).

The definitions of neurovascular orofacial pain (NVOP) by the ICOP [2], first described by us in 1997 as orofacial pain with vascular-type features [11], is typically tooth-located and aggravated by cold food or beverages, very similar to teeth affected by a carious lesion, except that these teeth were intact. We later, followed this with additional reports [1, 12, 13, 14], with our most recent study appearing in 2020 [15]. NVOP is characterized by strong pain (7–8 on Visual Analogue Scale (VAS)), pulsating and episodic. Pain may last minutes to hours, and up to 3 days [16]. Many cases are characterized by a high frequency, daily pattern of spontaneous pain or evoked by cold food ingestion.

ICOP highlights three distinct types of patients encountered in clinical practice, who appear to exemplify the intersection of headache and orofacial pain (OFP):

Type 1: Patients with headaches who also experience additional facial pain during their headache attacks, typically on the same side (ipsilateral) as the headache.

Type 2: Patients whose headache attacks have ceased but have been supplanted by facial pain attacks that mirror the former headache in quality, duration, and intensity, including the presence of associated symptoms.

Type 3: Patients without a history of headaches who develop new (*de novo*) OFP attacks that resemble primary headache types in terms of pain character, duration and intensity, with or without the associated symptoms typical of these headache types.

Only one study has field tested OFM [17]. In 44 patients with a facial presentation of migraine, type 1 accounted for 86.4%, followed by type 2 (11.4%) and type 3 (2.3%). Among 63 patients with Trigeminal Autonomic Cephalalgias (TACs) (including Cluster Headache), type 1 was 82.5%, followed by type 3 (17.5%), and none had type 2 phenotypes [17]. Although the proportion of type 3 among all patients with a facial presentation of primary headache has been reported [17], some patients may consult other specialties, such as otolaryngological practices because of the pain location [18, 19]. Furthermore, in a population-based study only one patient was identified with isolated facial migraine [20]. However, they admitted that this low rate could be due to a biased sampling; and those “having isolated facial pain without any other migraine symptoms could have been neglected” [20]. Therefore, the prevalence of type 3 orofacial pain may in fact be underestimated. Besides, type 3 patients can be difficult to demarcate from primary orofacial pain patients because of the lack of headache.

It seems that ICOP, for the purpose of “pure” classification includes under orofacial migraine (OFM) or neurovascular orofacial pain (NVOP) only patients in the third category, who have *de novo* pain exclusively in the facial region but with no head pain. Yet, in clinical practice we often see patients with a history of migraine, who suddenly develop severe tooth ache that does not respond to conventional dental treatment but treated successfully with antimigraine medications. Or patients who sometimes has “conventional head located” migraine, and on other occasions a migrainous toothache. These are often spontaneous or triggered. These patients should be included and studied in clinical studies of OFM or NVOP as has been reported [15, 16]. As in all classifications ICOP will develop and change as data is collected and published, hence all 3 types should be field-tested.

Recently, we reviewed the similar and unique features of NVOP and OFM and debated whether these two diagnostic entities should be merged, or their unique features justify the division into two separate diagnoses [21]. We concluded that the differences between NVOP and OFM are subtle, and their response to therapy seems to be similar [14, 19, 22, 23, 24]. Therefore, while classified under two separate entities, they contain many features in common, suggesting a possible overlap between the two. Consequently, their separation into two entities warrants further investigation [21]. With this aim the present study examined patients with facial presentations of neurovascular type pain that allowed the separation into two groups of NVOP and OFM. We compared their clinical features with the aim of reexamining whether they should preserve their two distinct division of two separate diagnoses, as classified under ICOP [2].

## 2. Materials and methods

### 2.1 Data collection

Data collection was retrospective with all patients interviewed and examined at the Orofacial Pain Clinic, Faculty of Dentistry, Hadassah-The Hebrew University, Jerusalem, Israel. Our clinic is a tertiary orofacial pain and headache center, and most patients were referred by general dentists, endodontic specialists, oral surgeons or ear, nose, and throat doctor (ENT) specialists. The data was drawn from a standardized intake form which includes demographics and a thorough pain history; using a standard form that includes questions such as: pain location, duration, intensity, *etc*. Most patients spoke Hebrew, but when necessary other languages were utilized such as Arab, English or Russian. Pain locations were charted on a diagram of the head and neck. Pain quality was evaluated by asking patients to describe their pain using terms such as electrical (including shooting), stabbing (including sharp), throbbing/pulsating, pressure or burning. These descriptors are routinely used in our clinic for quick pain quality assessments [25, 26]. Migraine-related symptoms such as photophobia, phonophobia, nausea and vomiting were also recorded. Additionally, cranial autonomic symptoms (CAS) like tearing and redness of the ipsilateral eye, nasal congestion/rhinorrhea, eyelid edema or localized swelling, increased sweating, flushing, ear fullness and ptosis and/or miosis during pain episodes were noted. Patients were specifically asked if pain woke them from sleep using a standardized question. Responses were carefully interpreted to confirm that pain was the cause of awakening, thereby excluding instances of random awakenings (*e.g.*, for drinking water or urination) where pain was coincidentally present but not the reason for awakening.

### 2.2 Inclusion criteria and pain diagnosis

Inclusion criteria consisted of all patients with neurovascular type pain that fitted the diagnoses of NVOP or OFM [2] and admitted to the clinic between 01 January 2018 and 31 December 2022. These are identical with the criteria in ICOP except that we were then collecting patients with associated headaches occurring concomitantly or non-concomitantly to the orofacial pain. All diagnoses were confirmed in the clinic and then re-examined following data tabulation and summary.

### 2.3 Clinical examination

All patients underwent a comprehensive extraoral and intraoral examination, which included an assessment of the masticatory apparatus. Intraorally, the examination aimed to rule out dental, periodontal and mucosal pathologies. Patients without prior dental radiographs were referred for radiographic evaluation to exclude clinically occult pathologies, utilizing full mouth periapical and/or panoramic radiographs.

### 2.4 Statistics

Data collected was tabulated and analyzed in SPSS (SPSS Statistics V29, IBM, USA). Missing data (shown in Table 1) for individual variables were coded within SPSS that adjusted the sample size for analysis accordingly. The null hypothesis was that there would be no differences in clinical features between OFM and NVOP with two-tailed α for significance set at 0.05. Associations between diagnosis and other nominal variables were analyzed with the Pearson Chi-squared (χ^2^) test. Relevant results are expressed as the mean and standard deviation. Following univariate analysis all statistically significant variables were entered and examined with a backward-stepwise logistic regression.

**Table 1.** Results, following univariate analysis all statistically significant variables were examined with a backward logistic regression.

Parameter		NVOP	OFM	Univariate *p*-value	Multivariate *p*-value
Sex (M:F)		6:34	6:29	NS	
Baseline VAS (±SD)		8 ± 1.9	9 ± 1.2	NS	
Mean Age (yr ± SD)		40.4 ± 13.6	38.5 ± 19.8		
	Male (yr)	35.2 ± 13.8	35.0 ± 14.4	NS	
	Female (yr)	41.3 ± 13.5	39.2 ± 20.9		
Laterality					
	Unilateral (n (%))	25 (64.1)	23 (65.7)	NS	
	Bilateral (n (%))	14 (35.9)	12 (34.3)		
	Missing	1	0		
Temporal Pattern					
	Continuous (n (%))	3 (7.5)	4 (11.8)	NS	
	Episodic (n (%))	37 (92.5)	30 (88.2)		
	Missing	0	1		
Pulsating (n (%))		16 (40.0)	17 (48.6)	NS	
Wakes (n (%))		10 (26.3)	18 (52.9)	0.020	NS
Missing		2	1		
Migraine Symptoms (n (%))		16 (41.0)	24 (68.6)	0.020	NS
Missing		1	0		
Headache					
	Concomitant (n (%))	12 (30.8)	22 (66.7)	0.001	NS
	Non-concomitant (n (%))	9 (23.1)	0		
	None (n (%))	18 (46.2)	11 (33.3)		
	Missing	1	2		
Mimics Toothache (n (%))		34 (85.0)	4 (11.4)	<0.001	<0.001
Missing		0	0		
CAS (n (%))		4 (10.3)	12 (36.4)	0.008	0.040
Missing		1	2		

*M: male; F: female; yr: year; NS: not statistically significant; SD: Standard deviation; CAS: cranial autonomic symptoms; NVOP: neurovascular orofacial pain; OFM: Orofacial migraine; VAS: Visual Analogue Scale. Missing: there were missing data in some of the outcomes measured explaining the percentages that are not a fit to the original sample size.*

## 3. Results

The cohort included 75 patients, 12 males and 63 females. Of this cohort 40 were diagnosed as NVOP and 35 as OFM. Further demographic details are shown in Table 1. The raw data were examined and based on the means and standard deviation univariate analysis was performed. Waking from sleep (NVOP 26.3%, OFM 52.9%, χ^2^ = 5.4, *df* = 1, *p* = 0.02), accompanying migraine symptoms (NVOP 41%, OFM 68.6%, χ^2^ = 5.6, *df* = 1, *p* = 0.02), the presence or absence of concomitant or non-concomitant headache (for %s see table, χ^2^ = 13.2, *df* = 1, *p* = 0.001), mimicking toothache (NVOP 85%, OFM 11.4%, χ^2^ = 40.4, *df* = 1, *p*≤ 0.001), and the presence of cranial autonomic symptoms (NVOP 10%, OFM 36.4%, χ^2^ = 7, *df* = 1, *p* = 0.008) were all statistically significantly different between NVOP and OFM. These parameters were entered into a regression analysis.

The final model of the multivariate analysis included cranial autonomic symptoms (CAS) and pain mimicking toothache. The Hosmer and Lemeshow goodness of fit test indicated a good fit (χ^2^ = 0.5, *df* = 2, *p* = 0.8). In this model the presence of autonomic signs increased the likelihood of an OFM versus NVOP diagnosis by an odds ratio of 5.6 with 95% confidence intervals (CI) at 0.98–36.2. Conversely, in neurovascular OFP that mimics toothache, the odds ratio of an NVOP versus an OFM diagnosis is increased to 33.9 (95% CI 7.9–146.5). The odds ratio is the predicted change in odds for a unit increase in the predictor. Results of analyses are shown in Table 1 and demonstrated in Fig. 1.

**Fig. 1.** Significantly distinctive features between Neurovascular Orofacial Pain (NVOP) and Orofacial Migraine (OFM), see Table 1. CAS: cranial autonomic symptoms.

## 4. Discussion

Orofacial migraine (OFM) and neurovascular orofacial pain (NVOP) are both recognized as migraine-related entities affecting the facial and orofacial regions, according to the International Classification of Orofacial Pain (ICOP). These conditions are associated with neurovascular-type headaches, including migraines and trigeminal autonomic cephalalgias (TACs), which share common characteristics such as the nature of the pain and accompanying symptoms like photophobia, phonophobia, nausea and vomiting. Cranial autonomic symptoms are also present and carry significant diagnostic value. The atypical orofacial location of these symptoms often leads to confusion and influences patients’ initial choice of healthcare consultation, making location a critical factor in the diagnostic process. The mechanisms underlying facial pain presentations of neurovascular headache disorders remain unclear [27]. One common hypothesis relies on the intracranial and extracranial innervation patterns of the trigeminal nerves. Therefore, the anatomical connection between the intracranial and extracranial fibers provides a route of how trigeminovascular activation of the dura extends to their extracranial counterpart, the V1 dermatome in the face.

The purpose of the present study was to examine the diagnostic characteristics of orofacial migraine (OFM) and (NVOP) and compare between the two. This, in order to reexamine whether they should be merged under a unifying diagnosis or conserve their distinct division of two separate diagnoses, as classified under ICOP [2]. The two groups demonstrated a similar male to female ratio of around 1 to 5, similar to that reported in previous studies [21]; with a much higher proportion of females than in migraine [28]. The age of patients, around 40, was also in accordance with previous studies [21] and is consistently higher than reported for typical migraine [28]. About one third of NVOP patients have bilateral pain with no difference versus OFM, and similar to that previously reported [21]. In both groups pain was largely episodic with a pulsating quality in about half of cases. NVOP contains fewer migrainous symptoms than OFM, and almost no cranial autonomic symptoms. Nevertheless, NVOP presents with migrainous characteristics, severe periodic pain and responds well to antimigraine medications [14, 15, 22, 29]. As clinicians the essence of diagnosis is therapy. NVOP’s similarities to migraine and specifically OFM are undeniable. It is important to appreciate that both facial pain disorders, with mixed migraine and trigeminal autonomic characteristics, are often misdiagnosed and repeatedly mistreated as dental or rhino nasal problems [16, 29, 30].

To balance this view, we found that patients with OFM reported significantly more awakening from sleep than NVOP patients. OFM patients also had significantly more migraine symptoms such as nausea and photophobia, as well as having threefold more cranial autonomic signs than NVOP patients (Table 1). Of special interest is the finding that OFM pain was concomitant with headache in about two third of cases, compared to only a third of cases of NVOP. In this study we included patients regardless of whether they had only facial symptoms or also those with a history of “conventional” migraine. These findings, especially the connection between OFM and the onset of headache, supports the need to include patients with headache in the diagnosis of patients with facial presentation of orofacial pain [21] in order to discriminate between OFM and NVOP. We therefore suggest expanding the definition of ICOP and include under OFM or NVOP, patients who have facial presentations of primary headache disorders with or without head pain.

The results of the univariate analysis suggest that NVOP is a different entity to OFM. The final model of the multivariate analysis only included cranial autonomic symptoms (CAS) and pain mimicking toothache. The fact that in the final model the pain in NVOP mimicked toothache is not surprising as this is part of its definition. Yet, one must appreciate that this toothache mimicking phenomenon is extremely rare in OFM (Table 1), which validates this finding as a distinguishing symptom between the two diagnoses. Pain that mimics toothache needs to be better described in order to indicate its unique features, not shared by OFM. Its main unique feature is that it is often evoked by cold food ingestion [14, 29] in addition to being spontaneous [21]. In many NVOP patients the application of a cold stimulus may detect more than one tooth with cold allodynia. The pain evoked is usually strong (VAS 8–9) and lasts up to 30 s after removing the cold stimulus. Root canal treatment may relieve the pain for a short while but pain may shift to another tooth [14, 29]. The pathophysiology of NVOP may be based on antidromic activation causing neurogenic inflammation (NI), similar to that which has been proposed in the past for migraine and/or cluster headache (CH). Following antidromic electrical nerve stimulation, NI has been demonstrated in the dental pulp of dogs, lower lip, and oral mucosa of rats [31]. Hypothesized neurophysiological and neurochemical substrates for migraine and cluster headache are therefore also present in the tooth. However, similar mechanisms are suggested also for OFM, not unlike the neurovascular mechanisms proposed for migraine and CH. Yet, the dental pulp is endowed by a unique anatomical structure. In the dental pulp, confinement by the surrounding dental hard tissues may lead to local build-up of pressure that contributes to intrapulpal nociceptor activation, resulting in strong pain similar to that of migraine headache confined by the skull. Aδ fibers have been shown to be sensitive to the increased intrapulpal pressure following plasma extravasation [32]. This neurogenic inflammation, similar in nature to pulpitis, should have resulted after some time in pulp necrosis, and pain should be abolished for a while [33]. However, homeostatic mechanisms limit pressure build up in the pulp following antidromic stimulation [32], probably by re-absorption into the circulation. This may explain clinical observations that in spite of pulpitis-like symptoms in the teeth of patients with NVOP, spontaneous pulp necrosis is rare.

The occurrence of CAS has been hypothesized to be related to pain intensity but in our series pain intensity in OFM and NVOP were similar. Possibly, the predominantly lower facial third location in NVOP whereas OFM is in the middle and lower thirds may account for the higher frequency of CAS in OFM. In TACs, the presence and type of CAS has been reported to be dependent on facial location [34]. However, the presence of facial pain in migraine increases CAS [16, 20]. The data suggest a more complex mechanism underlying the occurrence of CAS. The question on whether NVOP and OFM are separate entities is therefore not entirely substantiated in the multivariate analysis.

This study includes several limitations, such as its retrospective nature, limited information regarding various important aspects such as the rate of responsiveness to prophylactic and abortive migraine treatments, and a medium-sized cohort.

## 5. Conclusions

In conclusion, the diagnostic features of OFM and NVOP indicate that although there are unique features that allow for separating these as distinct diagnostic entities, there are many similarities between the two (Table 2). At present there seems justification to maintain the separate classifications of NVOP and OFM, particularly for research purposes. However, there is a need for larger, prospective studies to elucidate the relationship between NVOP and OFM.

**Table 2.** Common or unique features for orofacial migraine (OFM) and neurovascular orofacial pain (NVOP) based on the ICOP classification. Comments based on data of present study.

Feature	NVOP	OFM	Comments
Location	Intraoral and unilateral	Facial and/or oral and unilateral	NVOP may radiate to adjacent sites; bilateral cases up to a third
Time Course	1–4 hours, can extend beyond	4–72 hours (episodic), chronic (>15 days/month)	Duration overlaps, challenging distinct classification
Intensity	Moderate to severe	Moderate to severe	Similar intensity levels, complicating differential diagnosis
Symptoms	Pulsating, toothache-like pain predominant	Pulsating. Toothache-like pain less common	Toothache-like pain is a primary symptom in NVOP and a key differentiator from OFM
Associated Signs	Photo/phonophobia, nausea. CAS are rarer than in OFM	CAS. Nausea/vomiting, photophobia, phonophobia, aggravated by physical activity	NVOP requires only one associated sign for diagnosis; OFM typically involves multiple signs
Diagnostic Confusion	Often leads to initial non-specialist consultations due to location	Similar issue, but may include more direct headache features	Both conditions can lead to misdiagnosis due to atypical migraine-like location and presentation of symptoms
Prevalence of Concurrent Headache	Less common	More common, especially in chronic OFM	Headache co-occurrence supports differential diagnosis

*CAS: cranial autonomic symptoms.*
